# Advances in the study of phencyclidine-induced schizophrenia-like animal models and the underlying neural mechanisms

**DOI:** 10.1038/s41537-024-00485-x

**Published:** 2024-07-23

**Authors:** Dabing Li, Qiangwen Pan, Yewei Xiao, Kehui Hu

**Affiliations:** 1 Department of Physiology, School of Basic Medical Sciences, Southwestern Medical University, LuZhou, 646000 China; 2 Department of rehabilitation Medicine, SuiNing Central Hospital, The Affiliated Hospital of Chongqing Medical University, SuiNing, 629000 China

**Keywords:** Human behaviour, Schizophrenia

## Abstract

Schizophrenia (SZ) is a chronic, severe mental disorder with heterogeneous clinical manifestations and unknown etiology. Research on SZ has long been limited by the low reliability of and ambiguous pathogenesis in schizophrenia animal models. Phencyclidine (PCP), a noncompetitive N-methyl-D-aspartate receptor (NMDAR) antagonist, rapidly induces both positive and negative symptoms of SZ as well as stable SZ-related cognitive impairment in rodents. However, the neural mechanism underlying PCP-induced SZ-like symptoms is not fully understood. Nondopaminergic pathophysiology, particularly excessive glutamate release induced by NMDAR hypofunction in the prefrontal cortex (PFC), may play a key role in the development of PCP-induced SZ-like symptoms. In this review, we summarize studies on the behavioral and metabolic effects of PCP and the cellular and circuitary targets of PCP in the PFC and hippocampus (HIP). PCP is thought to target the ventral HIP-PFC pathway more strongly than the PFC-VTA pathway and thalamocortical pathway. Systemic PCP administration might preferentially inhibit gamma-aminobutyric acid (GABA) neurons in the vHIP and in turn lead to hippocampal pyramidal cell disinhibition. Excitatory inputs from the HIP may trigger sustained, excessive and pathological PFC pyramidal neuron activation to mediate various SZ-like symptoms. In addition, astrocyte and microglial activation and oxidative stress in the cerebral cortex or hippocampus have been observed in PCP-induced models of SZ. These findings perfect the hypoglutamatergic hypothesis of schizophrenia. However, whether these effects direct the consequences of PCP administration and how about the relationships between these changes induced by PCP remain further elucidation through rigorous, causal and direct experimental evidence.

Schizophrenia (SZ) is a chronic, severe mental disorder with heterogeneous clinical manifestations and unknown etiology. The SZ has a prevalence of 0.7–1.3% in the population and is ranked as the third leading cause of disability worldwide^1^. The clinical manifestations of SZ, including positive symptoms, negative symptoms, and cognitive deficits, are often difficult to alleviate and seriously affect human health^2–4^. Clinical studies on SZ have long been hindered by many unavoidable confounding factors, such as variations in disease subtypes and processes, as well as the influence of anti-SZ drugs, and animal experimental studies have long been limited by the poor reliability of SZ models and the lack of a clear mechanism of SZ pathogenesis in these models. A reliable animal model of SZ with clear pathogenic mechanisms is crucial for research on the pathology of and therapies for SZ^5^.

SZ is a disease unique to humans; thus, no animal model can recapitulate all of the symptoms of SZ. However, some behavioral changes seen in animal models are similar to those observed in SZ patients; thus, these animal models are valuable for research on the mechanism and treatment of SZ^6–8^. Current popular SZ models include genetically engineered models, brain injury models, pharmacological models, and neurodevelopmental disorder models^9,10^; each of these models has its own advantages and disadvantages in terms—some mainly recapitulate the positive symptoms of SZ and rarely exhibit negative symptoms^11^; others mainly recapitulate the negative symptoms of SZ. However, phencyclidine (PCP), a noncompetitive N-methyl-D-aspartate receptor (NMDAR) antagonist, was found to rapidly induce both the positive and negative symptoms of SZ as well as stable SZ-related cognitive impairment^1–3^, and this model has received widespread attention. In fact, the SZ pathogenetic hypothesis of the glutamatergic system deficiency was proposed based exactly on the observation that NMDAR antagonists such as PCP and MK801 lead to SZ-like behaviors^12^. However, the neural mechanism underlying PCP-induced SZ is not fully understood^13^. In this paper, we review research progress on the PCP-induced SZ-like animal model and summarize and speculate on the detailed neurobiological mechanisms by which PCP leads to persistent and stable SZ symptoms.

## Effects of PCP on psychological behaviors

PCP, a noncompetitive NMDA glutamatergic receptor antagonist commonly known as “angel dust”, was synthesized in 1956 and originally developed as a surgical anesthetic. Because of its low cost, high euphoric potential and hallucinogenic potential, PCP has been widely used as a recreational drug in Europe, the United States, and Asia since the 1970s. PCP has a wide range of pharmacological effects. At low concentrations (lower than the anesthetic dose), PCP binds to corresponding sites on the ion channels of NMDA receptors (NMDARs) and blocks Ca^2+^ inflow; however, at high concentrations (anesthetic doses), it also blocks receptors of many other transmitter systems, including the norepinephrine, serotonin, and dopamine systems^11^, as well as voltage-dependent sodium channels, potassium channels^14^, and even acetylcholine N subreceptors and other membrane proteins^15^. The affinity of PCP for NMDARs is 10 to 50 times greater than that of ketamine.

In healthy individuals, single or repeated administration of PCP below the anesthetic dose can cause a range of SZ-like symptoms, including hallucinations, paranoia and delusions, social withdrawal, emotional dullness, and cognitive impairment^11,16^. The symptoms caused by a single administration of PCP are similar to those observed in the early phases of SZ, while the symptoms caused by repeated PCP administration appear to be similar to those observed during the advanced phase of SZ^17,18^. Chronic PCP abusers are often misdiagnosed as having SZ.

Animal experiments have shown that systematic administration of PCP causes various behavioral abnormalities corresponding to human SZ symptoms, such as impairments in learning and memory, sociability locomotor activity, etc., in rodents^2,19,20^. These behavioral changes include sniffing, shaking the head, and frequent uprightness, which correspond to the positive symptoms of SZ^21,22^, and decreased sucrose consumption^23^ and social behavior^24^, which correspond to the negative symptoms of schizophrenia. PCP also causes impairments in delayed response tasks^25^ and working memory, such as reduced “detour fetch” behavior in monkeys^26^; and other impairments in attention/alertness, reasoning, and visual reversal learning^27,28^. Olfactory deficits^29^ as well as reduced prepulse inhibition (PPI), reflecting sensorimotor gating dysfunction, have also been observed in PCP-treated animals^30^. Consistent with these findings, acute systemic administration of MK801^31–33^, or ketamine^34–37^, another two noncompetitive NMDAR antagonists, also lead to similar SZ-like behaviors in rodents, zebrafish^38^, and monkeys^39^. Compared with PCP and ketamine, the NMDAR antagonist MK-801 has a higher selectivity and a greater inhibitory potency. Interestingly, drug-specific disturbances, especially difference between PCP and MK801 have been noticed^39,40^. Withdrawal from subchronic PCP but not MK-801 caused a delay-dependent impairment of working memory and reduced social interaction^40^.

Repeated administration of PCP has a long-lasting delayed effect on cognitive tasks. After long-term ( > 2 weeks) administration of PCP, monkeys exhibit deficits in “detour fetch” behaviors for at least 4 weeks following termination of PCP treatment^26^; deficits in associative motor learning measured by eyeblink conditioning in adult cynomolgus monkeys were still evident 18 months after two weeks of repeated PCP administration^41^. Apparently, these delayed effects of PCP may be due to its primary impact on the nervous system.

In general, the PCP-induced SZ-like animal model is considered the most reliable pharmacological model of SZ^42^. Compared to genetic or neurodevelopmental model of SZ, the PCP model is simple, cheap, and more feasible and convenient to reproduce. Behaviors resembling positive and negative symptoms and cognitive deficits can be exhibited immediately after the acute PCP administration.

Notably, the behavioral effects of PCP in rodents were widely affected by many experimental factors such as species (rat or mouse), dosages, and especially drug-delivery ways (by acute or subchronic administration). Though both acute and subchronic administration of PCP elicit various SZ-like behavioral deficits in rodents^19,43–45^, some studies failed to verify parts of these schizophrenia-like behaviors in rodents^45^, mostly attributing to difference in the dose used and the administration way among various studies. For example, although both acute and subchronic administrations of PCP increase locomotor activity, only a subchronic administration impairs working memory^22^. A previous study revealed that acute and subchronic PCP administration elicited a discrepant pattern of c-fos expression and differently affects neuronal activity in brain regions relevant to SZ. Acute PCP increased c-fos expression in many cortical regions while subchronic one increased c-fos expression in few regions such as the retrosplenial cortex, thalamus, hippocampus, and supramammillary nucleus^22^. Another mechanism account for the different behavioral effect of acute and subchronic PCP may be that different aspects of mental processes are disturbed by different paradigms of PCP applications—acute PCP administration impaired the inhibitory control, whereas the chronic one decreased the processing speed^18^. Several examples of PCP administration and its behavioral effects were illustrated in Table 1.

**Table 1:** Several examples of PCP administration and its behavioral effects.

Species/Ref.	PCP dosage and administration	Behavioral Effect	
		positive/negative	cognition
Mice^19^	2.5 mg/kg, 15 d 10 mg/kg at 16 d, subchronic/ s.c.	↑locomotor activity ↑anxiety behavior	↓memory performance PPI deficits
Mice^20^	10 mg/kg, acute/ i.p	↑locomotor activity	
Mice^2^	10 mg/kg, 10 d subchronic/s.c	↑locomotor activity ↑immobility time in FST	↓memory performance
Rat^3^	10 mg/kg, 3 times (7d, 9d, 11d)/S.C. +socially isolated (post-weaning)	↑locomotor activity	↓social recognition ↓PPI ↓reveral learning
Rat^126^	2 mg/kg, 3 times (7d, 9d, 11d)/S.C. +2 h restrain + 20 min forced swimming (34 d)	↑anxiety-like behavior locomotor activity (−)	↓novel object recognition memory ↓hippocampal LTP
Rat^29^	3.3 mg/kg, in adult or 10 mg/kg at 7d, 9d, 11d in postnatal rats, S.C.	↓olfactory sensitivity	
Mice^57^	10 mg/kg, 10 d subchronic/s.c		↓novel object recognition memory
Mice^44^	10 mg/kg, 10 d subchronic/s.c		↓ novel object recognition memory
Rat^2^	10 mg/kg, 10 d S.C.	↑locomotor activity ↑forced swimming	↓novel object recognition memory
Rat^28^	1.5 mg/kg, 4 d subchronic/s.c		↓visual reveral learning response latency or trials(−)
Mice^142^	2, 5, 10 mg/kg, 7 d subchronic/s.c 24 h wash out	swim speed (−)	10 mg/kg ↓ space learning and memory 2 mg/kg (−)

(-): no changes, ↑ increased/enhanced, ↓ decreased/weakened, *PCP* Phencyclidine, *PPI* Prepulse inhibition, *d* days, *s.c.* Subcutaneous, *i.p.* Intraperitoneal, *min* minutes, *LTP* Long-term potentiation, *FST* Forced swimming test, *Ref*. references.

## Effects of PCP on neurotransmitters and metabolism in the brain

Experimental animal studies have shown that chronic and repeated administration of PCP at a low dose produces specific metabolic and neurochemical changes in the rodent brain that are strikingly similar to the changes in the brains of SZ patients^46^. A single intraperitoneal injection of PCP increases the levels of glutamate, dopamine, 5-hydroxytryptamine (5-HT), and norepinephrine and decreases extracellular concentrations of gamma-aminobutyric acid (GABA)^47^ in the medial prefrontal cortex (mPFC) of rats^48^, whereas in several other brain regions^49^, dopamine levels are elevated after PCP administration. Glutamate receptor agonists can inhibit the abovementioned changes induced by PCP and block the increase in locomotion induced by PCP^49^. Consistent with this, acute systemic administration of MK801 also increases the release of several neurotransmitters, including glutamate^50^, dopamine^49^, 5-HT^51^, and acetylcholine^52^, in the rodent prefrontal cortex (PFC). Excessive glutamate release in the PFC may be caused by hypofunction of NMDAR and play a key role in the development of PCP-induced SZ-like symptoms, while PCP-induced NMDAR hypofunction was also considered to be related to several symptoms of cognitive deficits in SZ^53–56^.

In PCP-treated mice, decreased brain-derived neurotrophic factor (BDNF) levels and a reduced ratio of phosphorylated TrkB (p-TrkB) to TrkB in the PFC and hippocampus are accompanied by cognitive deficits^57–59^. (R)-Ketamine can ameliorate PCP-induced cognitive deficits by activating BDNF-TrkB signaling in the brain^57^. There is also an association between peripheral BDNF levels and cognitive deficits in patients with SZ^60–63^. BDNF has a complex and rare genetic structure. Earlier studies mainly observed changes of BDNF-TrkB signaling in PCP models, indicating the direct effects of PCP on neurotrophic signaling pathway. However, a recent study found that the combination of the subtype of BDNF-E6 knockout, and adverse environmental stress during development can cause SZ-like behavior in mice^64^. Given the critical role of BDNF-TrkB signaling in neuroplasticity, it suggests that abnormalities in this signaling pathway may be the key genetic factor of SZ pathogenesis.

In addition, PCP increases NO levels in the PFC and ventral hippocampus (vHIP) in animals^9^, indicating that NO seems to be a key signaling molecule in SZ. In subchronic phencyclidine (scPCP)-treated mice, the numbers of inhibitory interneurons labeled with parvalbumin and the levels of PSD95 in the PFC and vHIP are both significantly reduced^44^, and polysialic acid neural cell adhesion molecule (PSA-NCAM) levels in the striatum are significantly decreased^13^. Enhanced inflammation and oxidative stress can be observed in the PFC of PCP-induced SZ-like mouse model^2^.

Clinical studies have demonstrated that glutamate levels are increased in hippocampus of SZ patients, which possibly activated hippocampal neurons and correlated well with the severity of SZ^65^. Abnormally excited hippocampal neurons may directly or indirectly activate dopaminergic and 5-HT neurons in the midbrain and enhanced release of monoamine transmitters^66,67^, which involving in mediating the PCP-induced SZ-like symptoms.

## Effects of PCP on Neuroglia, inflammatory and oxidative responses

Astrocyte is the most abundant cell type in the mammalian brain. Growing evidence indicates the astrocyte is the key regulator of brain energy metabolism^68^. Astrocytic glutamate uptake triggers aerobic glycolysis and leads to glucose uptake and lactate release to regulate neuro-energetic coupling. In addition, the synthesis of glutamate and GABA in neurons is closely connected to astrocyte metabolism. Administration of PCP (10 mg/kg/day; 7 days) increased significantly the expression of the astrocytic Glu transporter GLT-1/EAAT2 and Glu uptake, in both rats’ brain slices and neuron/astrocyte co-cultures, thereby reducing extracellular^69^. Another NMDAR antagonist MK801 (0.5 mg/kg/day, 6 days) is reported to increase first, and then decrease later, the levels of glutamate in frontal and cingulate cortices, mimicking the increased glutamate/glutamine activity found in drug-naive patients with first episode SZ and the transition to lower glutamatergic function seen in chronic SZ^70^.

Through ex vivo and in vivo two-photon astrocyte imaging, a recent study found that brief, subcellular inputs of GABA and glutamate results in widespread, long-lasting astrocyte Ca^2+^ responses^71^, supporting the hypothesis of astrocyte-neuron communication across slow, network-level spatiotemporal scales^72–74^, i.e., local, transient neurotransmitter inputs cause changes of broad cortical astrocyte networks over a minutes-long time course^75–78^. Thereby the disturbance in astrocytic functions may be of significance in malfunction of the glutamine-glutamate-GABA cycle and the pathogenesis of animal model of SZ caused by NDMAR blockade.

Astrocytes are also neurotoxic reactive cells and regulate brain response to injury, stimulus, and neurodegenerative disease^79^. Neuroinflammatory microglia can secrete cytokines to induce neurotoxic reaction of astrocytes to causes neuronal death^80^. Notably, Chronic PCP administration in mice (20 mg/kg, i.p., 7 days) up-regulated proinflammatory cytokine interleukin-1β and induced astrocyte and microglial activation in both the cortex and hippocampus^81^; PCP increased cytokines including p-p65, p-IκBα, p-p38 in the prefrontal cortex of mice^2^, indicating a significant role of neuroinflammation in the pathophysiology of SZ model of PCP. Perhaps these changes of neuroglia particularly astrocytes and the inflammatory responses as target of PCP might affect the normal functions of ventral HIP-PFC pathway, thereby contribute to PCP-induced SZ-like behaviours.

Recent evidence also reveals that oxidative stress plays a critical role in the etiology of SZ. Hippocampal oxidative stress was verified by increased nitrotyrosine, a protein marker of oxidative stress, in a PCP (50 mg/kg, subcutaneously) rat model of SZ^82^. In wild-type mice, PCP also resulted in an early increase of dinucleotide phosphate oxidase activity, mitochondrial burdens, and decreases of mitochondrial superoxide dismutase and glutathione peroxidase activities 4 days later^83^. Interestingly, recent study found that betaine and rographolide can improve SZ-like behaviors through inhibition of both inflammation and oxidative stress in PCP-induced models of SZ^2,84^.

In brief, enhanced inflammation and oxidative stress observed in PCP-induced SZ models align with findings in SZ patients and demonstrate a more complex interaction within the disease model. Whether these effects direct the consequences of PCP administration and how about the relationships between neuroinflammation, oxidative stress, and the neurotransmitter changes induced by PCP remains further elucidation.

## Neuronal targets of PCP

Studies conducted in the 80’ and early 90’ in last century has ever mentioned that PCP receptors are the targets for PCP. However, further studies indicate that PCP mainly exerts its affects by targeting the NMDAR. Early studies identifying distribution of PCP “receptors” (celluar targets) by autoradiography using [^3^H]TCP, a PCP marker with a similar conformation as PCP. Studies in rats and guinea pigs revealed that^85^ the highest levels of PCP receptors are found in the forebrain, especially in the superficial layers of the cerebral cortex and hippocampus and in the molecular layer of the dentate gyrus; however, in the amygdala, thalamus, olfactory node, geniculate nucleus, and deeper layers of the cortex, the density of PCP receptors is moderate. The lowest density of PCP receptors is found in the septal regions, hypothalamus, pontine medulla and cerebellum. Differences in the presynaptic or postsynaptic distribution of PCP receptors in the hippocampus and striatum were measured by combined application of [^3^H]TCP and selective lesioning of neuronal bodies or afferent fibers^86^. The results showed that NMDA and PCP receptors are located predominantly on the cell bodies of intrinsic neurons in these regions and that fewer than 10% of PCP receptors are autoreceptors at the terminals of glutamatergic afferent fibers. In brief, the distribution of PCP “receptors” suggests that the prefrontal cortex and hippocampus may be important target areas of PCP. Consistently, clinical studies have long suggested that structural or functional impairment of these two regions is commonly involved in SZ^87^.

Direct injection of PCP into the local mPFC does not activate neurons in this region^88^, and application of PCP to brain slices does not result in a significant increase in the EPSC amplitude of mPFC neurons^89^. However, systemic administration of PCP (i.p.) produces sustained, tonic, and mild excitation rather than epileptiform firing of mPFC neurons in freely moving rats, which occurs almost simultaneously with increased locomotion and stereotypic behaviors^90^; acute systemic administration of MK801, an NMDAR antagonist with a similar mechanism of action as PCP, also significantly increases the firing frequency and c-fos mRNA expression in PFC pyramidal neurons in animals^90–93^. In anesthetized rats, systemic administration of PCP or MK801 also induces excitation of mPFC neurons^90,94^, suggesting that the excitatory effect of PCP on the mPFC is not secondary to the altered arousal levels induced by increased locomotion in animals. Consistently, inhibition of MK801-induced tonic excitation of mPFC neurons ameliorates motor abnormalities^95^. Notably, systemic administration of methamphetamine, a typical psychostimulant, fails to elicit similar activation of mPFC neurons in animals despite inducing hyperlocomotion^96^, suggesting that distinct neural circuits mediate PCP- and methamphetamine-induced behaviors in SZ-like models. In addition, the atypical antipsychotic drug clozapine inhibits PCP-induced c-fos expression in the mPFC^91^.

It has been proposed that tonic activation of the PFC by excitatory inputs from the hippocampus is crucial in the development of PCP-induced psychosis^96^. Hippocampal hyperfunction may be the main etiology of positive symptoms of SZ^65^. Excitation of hippocampal neurons can directly or indirectly activate dopamine- and 5-HT-secreting cells in the midbrain, leading to enhanced release of monoamine transmitters^66,67^, which in turn causes related positive symptoms; direct targeting of the hippocampus by deep brain stimulation (DBS) reverses abnormalities in dopaminergic neuronal activity in animals^97^, which supports the idea that hippocampal hyperfunction occurs in SZ.

PFC dysfunction may be closely associated with the negative symptoms and cognitive deficits related to SZ^98^, such as working memory decline^99^ and deficits in sensorimotor gating and attention^100^; hypofrontality in SZ patients has been revealed by positron emission tomography (PET) during verbal learning tasks, indicating the complex role of PFC in SZ (more complex than a positive or negative effect)^98^. In addition, a clinical study reported that patients with SZ show reduced hippocampal activity during word encoding and recognition^101^, suggesting the diverse hippocampal dysfunctions in SZ^98^. However, the PFC and hippocampus are considered potential targets for neuromodulation and the treatment of SZ^97^, and the CA1 subregion of the vHIP is the most promising target brain region^102^.

## Circuitary targets of PCP

There are several hypotheses regarding the mechanism by which PCP decreases NMDAR function and triggers subsequent changes.

### Ventral HIP-PFC pathway

Anatomical studies have shown that there are extensive monosynaptic, unidirectional, and ipsilateral excitatory projections from the hippocampus to the PFC in primates and rodents. These projections originate from the CA1 subregion of the vHIP and form monosynaptic connections with pyramidal and GABAergic neurons in multiple subregions of the PFC. Functional studies have demonstrated that these projections are involved in a variety of cognitive processes, such as gating of sensorimotor information^103^ and fear conditioning^104^, and are therefore associated with the development of a variety of neurological and psychiatric disorders. The reduced hippocampal volume in depression and PTSD patients suggested that deficient hippocampus-mediated inhibition of the PFC may underlie emotional disorders^104^.

**Fig. 1:**
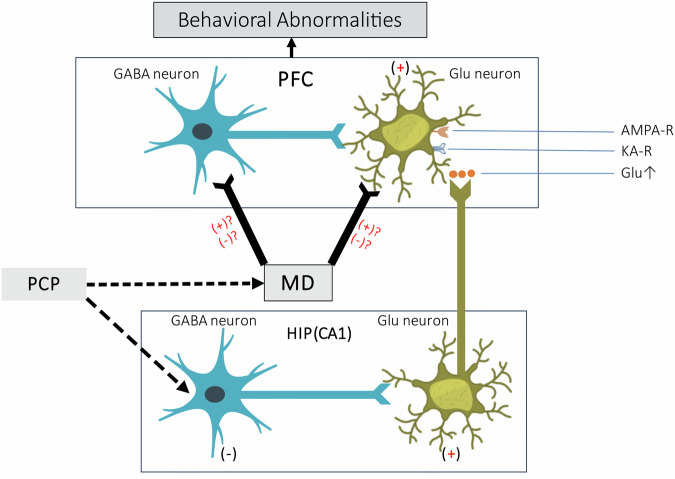
The ventral HIP has a dense glutamatergic projection to the PFC. PCP induces inhibition of tonic GABA inputs due to the dense distribution of NMDAR on GABA neurons. NMDAR has a sparse distribution on Glu neurons. Dotted arrows denote an indirect influence or indirect connection. PFC prefrontal cortex, PCP phencyclidine, GABA gamma-aminobutyric acid, Glu glutamatergic, AMPA-R α-amino-3-hydroxy-5-methyl-4-isoxazole-propionic acid receptor; KA-R kainic acid receptor, HIP hippocampus, MD mediodorsal nucleus of the thalamus. ↑, increased; (+), excitation; (−), inhibition.

Clinical studies have revealed increased activation of the PFC in patients with first-episode SZ^105^ and after ketamine administration in healthy individuals, suggesting increased glutamate release in the PFC^106^. Experimental animal studies have revealed that^90^ acute systemic administration of PCP results in a significant increase in the extracellular glutamate concentration in the mPFC, as well as prolonged excitation of mPFC neurons and increased locomotion in free-moving animals. The temporal changes in mPFC neuronal activation parallel the animals’ behavioral changes over time, suggesting that the behavioral abnormalities induced by systemic administration of PCP may be caused by overactivation of mPFC neurons. Curiously, however, local injection of PCP directly into the PFC does not increase the firing of local neurons^90^, whereas local infusion of PCP into the vHIP induces tonic activation of mPFC neurons and enhanced spontaneous activity in animals^88^. Systemic administration of PCP appears to stimulate the overactivation of mPFC neurons via excitatory inputs from brain regions outside the mPFC. Accordingly, an important hypothesis regarding the involvement of NMDAR hypofunction in pharmacological models of SZ has been proposed; this hypothesis postulates that by inducing excessive and pathological excitation of ventral hippocampal-prefrontal projections, PCP may lead to SZ-related symptoms^88,90^ (see Fig. 1). Studies supporting this hypothesis have shown that hippocampal neurons projecting directly to the mPFC are more susceptible to activation by NMDAR antagonists^88^, although why this hippocampal neuronal subgroup is particularly sensitive to excitation induced by PCP or MK801 is unclear. The distinct distributions of NMDARs on these projection neurons and the corresponding different local circuits within the vHIP may contribute to this phenomenon^96^.

Optogenetic studies have shown that inhibition of CA1 neuronal activity in the vHIP alleviates eyeblink conditioning deficits, an associative learning task, in a PCP-treated rat model of SZ^107^. This causal evidence suggests that ventral hippocampal neurons may be important targets of PCP in recapitulating SZ symptoms. However, the specific cellular and circuitry mechanisms through which PCP activates hippocampal neurons and thus PFC neuronal activity are still unclear.

How does PCP induce pathologic excitation of vHIP-PFC projections? Relevant studies suggest that the composition^108^ and number (more abundant) of NMDARs on GABAergic neurons are distinct from those on pyramidal cells in the CA1 and CA3 subregions of the hippocampus; this difference may result in preferential inhibition of GABAergic neurons rather than pyramidal neurons when PCP is administered^109^ and in turn leads to disinhibition of connected pyramidal neurons. For example, the NR2C and NR2D subunits are prominently expressed in subsets of interneurons, while the NR2A and NR2B subunits are prominently expressed in pyramidal neurons^110^. Therefore, the relatively enrichment of NMDARs or differences in NMDAR subunits on GABAergic neurons may be the basis for PCP-induced activation of vHIP-PFC projections. Consistent with this hypothesis, GAD65 and GAD67 levels are reduced in the hippocampus of patients with SZ^111^, suggesting that dysfunction of GABAergic interneurons^112^ is responsible for controlling the excitatory state of pyramidal cells^102^. In addition, direct injection of α-amino-3-hydroxy-5-methyl-4-isoxazole-propionic acid (AMPA) and kainic acid (KA) receptor antagonists into the mPFC results in the inhibition of mPFC neuron excitation induced by systemic administration of PCP or MK801, whereas local application of acetylcholine muscarinic and nicotinic receptor antagonists have little effect^94^, suggesting that the effect of vHIP-PFC projections in activating mPFC neurons is mediated by AMPA and KA receptors within the mPFC.

Theoretically, PCP can also directly block NMDARs on GABAergic interneurons in the PFC, thereby inhibiting GABAergic neurons in the PFC and in turn lead to an increase in cortical activity and γ-band oscillations^113,114^. However, microiontophoretic application of PCP directly into the PFC exerts little influence on the spontaneous firing activity of PFC neurons^90^. The hippocampus may be much more sensitive to PCP than other brain structures according to relevant studies^109^.

Taken together, these findings support the hypothesis that PCP preferentially inhibits GABAergic neurons in the vHIP, which in turn leads to disinhibition of hippocampal pyramidal cells as well as excessive and pathological activation of vHIP-PFC projections. Moreover, changes in astrocyte as target of PCP might also directly or indirectly affect the normal functions of vHIP-PFC pathway and contribute to PCP-induced SZ-like behaviours.

Series evidence indicate that tonic activation of vHIP-PFC projection is responsible for some of the positive symptoms such as hyperlocomotion and stereotypic behaviors, through long-lasting exciting pyramidal cells in the mPFC of PCP-induced model, though mPFC itself does not directly provoke locomotor activity^90^. Pathological activation of vHIP-PFC projection may also be implicated in some of the negative symptoms such as social withdrawal and avolition^115^, via reduction of dendritic spines and receptors in the mPFC of PCP-induced model of SZ. Moreover, it is comprehensible that excessive activation of vHIP-PFC pathway is involved in the development of cognitive malfunction in this model given the widely recognized pivotal roles of the PFC in attention, planning, working memory and emotion processing. Besides, PCP might induce positive and negative symptoms in rodents through VTA mesolimbic pathway and VTA mesocortical pathway, respectively, which are all activated indirectly by PCP administration and in turn excitation of PFC-VTA Pathway (see below).

Notably, bilateral excitotoxic damage to the vHPC are commonly used to establish developmental models of SZ^116^. In addition, Sotres-Bayon F. et al reported that pharmacological inactivation of the vHPC increases the activity of pyramidal neurons and decreases the activity of interneurons in the PrL^104^, suggesting that the vHIP exerts an inhibitory effect on the PFC, which seems to contradict the hypothesis that disinhibition of hippocampal pyramidal cells excites PFC neurons. In summary, rigorous, causal and direct evidence is needed to verify the assumption that PCP induces SZ pathogenesis through excessive and pathological activation of vHIP-PFC projections.

### PFC-VTA pathway

**Fig. 2:**
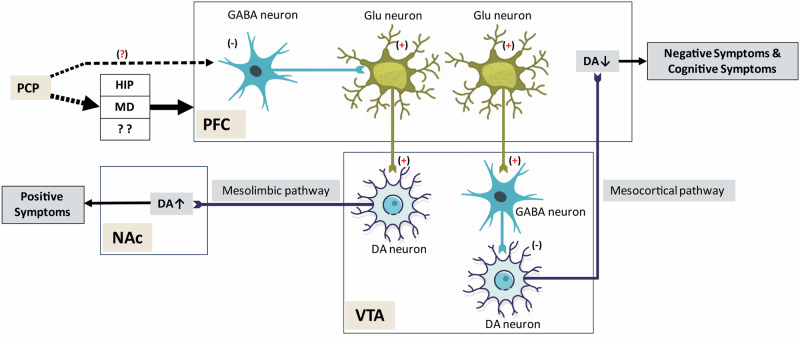
PCP Acts on PFC-VTA Pathway. PCP increases glutamatergic inputs from PFC to VTA & activate VTA-NAc projection & inactivate VTA-PFC projection, respectively. Solid arrows denote a direct influence or direct connection; Dotted arrows denote an indirect influence or indirect connection. PCP phencyclidine, PFC prefrontal cortex, HIP, hippocampus; MD, mediodorsal nucleus of the thalamus; GABA, gamma aminobutyric acid; DA, dopamine; NAc, nucleus accumbens; VTA, ventral tegmental area. (+), excitation; (−), inhibition; ↑, increased; ↓, decreased.

Another hypothesis postulates^42^ that by blocking NMDARs on gamma-aminobutyric acid (GABA) interneurons in the PFC, PCP leads to disinhibition of the subgroup of PFC glutamatergic neurons projecting to the VTA, which in turn leads to overactivation of the mesolimbic dopaminergic pathway and subsequently to positive symptoms similar to those observed in SZ patients. In addition, researchers have hypothesized that disinhibition of PFC glutamatergic neurons projecting to the VTA leads to overactivation of VTA GABAergic interneurons and, in turn, inhibition of mesocortical dopaminergic pathways and decreased dopamine release in the PFC, which are responsible for both the negative and cognitive symptoms of SZ (see Fig. 2). It is surprising that PFC glutamatergic projections to the VTA differentially induce excitation of the dopamine (DA) mesolimbic system and inhibition of the DA mesocortical system. It is worthwhile to clarify whether and how these opposite effects occur.

In addition, previous studies have confirmed that chronic administration of PCP induces upregulation of NMDARs and damage to cortical neurons in the rodent forebrain^117,118^. A recent hypothesis suggested that the abovementioned changes may lead to disturbances in cortico-striatal communication. Reduced cortical inputs to the striatal nucleus may affect synaptogenesis or synaptic plasticity in subcortical structures^13^.

### Thalamocortical pathway

Systemic administration of PCP also activates medial thalamic (MD) neurons^91^ and increases the activity of thalamocortical networks by preferentially blocking NMDARs in the reticular nucleus of the thalamus^119,120^. Approximately one-third of MD neurons exhibit tonic excitation in response to systemic administration of PCP^121^, suggesting that the MD may also be a source of PCP-induced excitatory inputs to the mPFC. However, MD axons terminate mainly on GABAergic interneurons, rather than pyramidal neurons, in the mPFC^122^; MD stimulation mainly increases c-fos expression in GABAergic neurons in the mPFC^123^ and suppresses the excitation of mPFC neurons in response to hippocampal stimulation^124^. Therefore, MD inputs have an inhibitory rather than an excitatory effect on mPFC pyramidal neuronal activity.

## Limitations of PCP-induced animal models of SZ and related improvements

An excellent animal model needs to have three characteristics: Face Validity based on human disease characterization; Construct Validity based on human pathogenesis; Predictive Validity to validate drug efficacy. So far, it has been a major challenge in current research to model SZ, as well as other mental illness, since that animals do not exhibit or it is hard to measure the higher mental functions or states of the tested animal. Thus animal models of SZ, whether pharmacological, genetic, or neurodevelopmental mimicking, failed to fully reflect the mental state of SZ observed in humans. Moreover, manifestations of SZ are often chronic and started mostly from puberty, whereas administration of PCP mainly resembles acute psychosis, not a permanent mental state. Another limitation of PCP model is that it provides limited value in explaining the etiopathology of SZ.

With the deeper understanding of SZ pathogenesis, the interaction of genetic factor and environmental risk factor are recognized to ultimately leads to SZ (“double whammy” hypothesis). To resemble more obvious and representative behaviors of SZ, improvements have been made to reproduce SZ-like model with PCP recently^3,125–127^. These improvements come in two ways. The first is the administration of PCP early during the prenatal or perinatal period to interfere with early neurodevelopment and thereby mimic the developmental etiology of SZ. Early administration of PCP in the pre- or postnatal period in rodents is more likely to induce long-lasting SZ-related behaviors. The second method is the combination of PCP administration with other interventions related to SZ model reproduction. For example, after PCP was administered in the postnatal period, mental stimulation was administered during adolescence^19,128–130^;. The hypothesis related to this combination is that PCP provides the “first hit” to the developing brain to trigger vulnerability to future stress and that a subsequent “second hit” (stressful stimuli^126^ or social isolation^3^) at a specific time triggers the emerging of schizophrenia-like state in animals. These improvements have extended the application of the PCP model of SZ and are supported by similar studies with ketamine to induce neurodevelopmental toxicity^131^.

## Questions and expectations

Pharmacological treatment of SZ has not progressed significantly in more than half a century^132^. Currently, targeting monoamine receptors is still the main strategy in clinical practice, yet the negative symptoms and cognitive symptoms of SZ are not effectively ameliorated by conventional antipsychotic medications. Thus, new conceptualizations and therapeutic approaches for SZ are urgently needed based upon glutamatergic theories.

The NMDARs, which are located throughout the brain system and blocked in the PCP-induced model of SZ, have been recognized to play an important role in the nondopaminergic pathophysiology of SZ. NMDARs are also located on neural circuits that regulate DA release, suggesting that glutamatergic system deficits may lead to dopaminergic system dysfunction in SZ. Since direct agonist of NMDAR may cause excitotoxic damage to neurons, therapeutic researches have focused on indirect NMDAR agonists. However, drugs that stimulate NMDAR-mediated neurotransmission, including glycine-site agonists, D-serine-based compounds and glycine transport inhibitors demonstrated inconsistent results in preclinical studies^8,133^, though agents such as agonists of mGluR2, 3, can decrease resting glutamate levels and reverse potential disruption in firing patterns within prefrontal cortex^134^. The disappointing results of targeting NMDARs with indirect receptor agonists may be attributed to the lack of a clear understanding of the neuronal and circuitry mechanisms involved in the dysfunction of the glutamine system in SZ^8^. Moreover, promising therapeutic effect of NMDAR modulators require that the potential risks posed by the use of such agents are excluded^135^. Interestingly, an NMDAR antagonist with low affinity and has been approved for the treatment of Alzheimer’s disease, contributes to the alleviation of negative and cognitive symptoms and attention deficits in SZ^5,136–138^.

In addition, electrical neuromodulation of activities of specific brain nuclei or subregions such as vHIP have been explored for SZ therapy in recent years^100,139,140^. Clinical DBS studies in patients with refractory SZ have reported preliminary yet promising results^141^. However, successful targeted interventions must be based also on a precise and thorough understanding of the abnormal functional circuits involved in SZ. Therefore, it is necessary to confirm whether the vHIP and its specific efferents mediates PCP-induced SZ-related symptoms and how about the role of GABAergic neurons in this process, as there are multiple types of excitatory and inhibitory neurons and extensive efferent projections in the vHIP. Such studies will aid the identification of promising targets and strategies to treat SZ.

## References

[1] Adell, A. Brain NMDA receptors in schizophrenia and depression. *Biomolecules* **10**, 947 (2020). 32585886 10.3390/biom10060947 PMC7355879

[2] Wang, X., Liu, J., Dai, Z. & Sui, Y. Andrographolide improves PCP-induced schizophrenia-like behaviors through blocking interaction between NRF2 and KEAP1. *J. Pharm. Sci.* **147**, 9–17 (2021). 10.1016/j.jphs.2021.05.007 34294378

[3] Hamieh, A. M., Babin, D., Sable, E., Hernier, A. M. & Castagne, V. Neonatal phencyclidine and social isolation in the rat: effects of clozapine on locomotor activity, social recognition, prepulse inhibition, and executive functions deficits. *Psychopharmacology* **238**, 517–528 (2021). 33169202 10.1007/s00213-020-05700-y

[4] Ang, M. J., Lee, S., Kim, J.-C., Kim, S.-H. & Moon, C. Behavioral tasks evaluating schizophrenia-like symptoms in animal models: a recent update. *Curr. Neuropharmacol.* **19**, 641–664 (2021). 32798374 10.2174/1570159X18666200814175114 PMC8573744

[5] Kikuchi, T. Is memantine effective as an NMDA-receptor antagonist in adjunctive therapy for schizophrenia? *Biomolecules* **10**, 1134 (2020). 32751985 10.3390/biom10081134 PMC7466074

[6] Bialon, M. & Wasik, A. Advantages and limitations of animal schizophrenia models. *Int. J. Mol. Sci.* **23**, 5968 (2022). 35682647 10.3390/ijms23115968 PMC9181262

[7] Wu, Q., Huang, J. & Wu, R. Drugs based on NMDAR hypofunction hypothesis in schizophrenia. *Front. Neurosci.-Switz.* **15**, 641047 (2021). 10.3389/fnins.2021.641047 PMC8072017 33912003

[8] Wolf, D. H. et al. Effect of mGluR2 positive allosteric modulation on frontostriatal working memory activation in schizophrenia. *Mol. Psychiatr.* **27**, 1226–1232 (2022). 10.1038/s41380-021-01320-w PMC9018886 34667261

[9] Morales-Medina, J. C., Aguilar-Alonso, P., Di Cerbo, A., Iannitti, T. & Flores, G. New insights on nitric oxide: Focus on animal models of schizophrenia. *Behav. Brain Res* **409**, 113304 (2021). 33865887 10.1016/j.bbr.2021.113304

[10] Perez-Palomar, B., Erdozain, A. M., Erkizia-Santamaria, I., Ortega, J. E. & Meana, J. J. Maternal Immune Activation Induces Cortical Catecholaminergic Hypofunction and Cognitive Impairments in Offspring. *J. Neuroimmune Pharm.* **18**, 348–365 (2023). 10.1007/s11481-023-10070-1 PMC10577104 37208550

[11] Thornberg, S. A. & Saklad, S. R. A review of NMDA receptors and the phencyclidine model of schizophrenia. *Pharmacotherapy* **16**, 82–93 (1996). 8700797 10.1002/j.1875-9114.1996.tb02920.x

[12] Bunney, B. G., Bunney, W. E. & Carlsson, A. *Schizophrenia and Glutamate in Psychopharmacology: The Fourth Generation of Progress*. 1205 (Raven Press, 2005).

[13] Wang, C., Inselman, A., Liu, S. & Liu, F. Potential mechanisms for phencyclidine/ketamine-induced brain structural alterations and behavioral consequences. *Neurotoxicology* **76**, 213–219 (2020). 31812709 10.1016/j.neuro.2019.12.005

[14] ffrench-Mullen, J. M. & Rogawski, M. A. Interaction of phencyclidine with voltage-dependent potassium channels in cultured rat hippocampal neurons: comparison with block of the NMDA receptor-ionophore complex. *J. Neurosci. : Off. J. Soc. Neurosci.* **9**, 4051–4061 (1989). 10.1523/JNEUROSCI.09-11-04051.1989 PMC6569947 2555461

[15] Oswald, R. E., Bamberger, M. J. & McLaughlin, J. T. Mechanism of phencyclidine binding to the acetylcholine receptor from Torpedo electroplaque. *Mol. Pharm.* **25**, 360–368 (1984). 6727862

[16] Newcomer, J. W. et al. Ketamine-induced NMDA receptor hypofunction as a model of memory impairment and psychosis. *Neuropsychopharmacol.: Off. Publ. Am. Coll. Neuropsychopharmacol.* **20**, 106–118 (1999). 10.1016/S0893-133X(98)00067-0 9885791

[17] Neill, J. C., Harte, M. K., Haddad, P. M., Lydall, E. S. & Dwyer, D. M. Acute and chronic effects of NMDA receptor antagonists in rodents, relevance to negative symptoms of schizophrenia: a translational link to humans. *Eur. Neuropsychopharmacol. : J. Eur. Coll. Neuropsychopharmacol.* **24**, 822–835 (2014). 10.1016/j.euroneuro.2013.09.011 24287012

[18] Thomson, D. M., McVie, A., Morris, B. J. & Pratt, J. A. Dissociation of acute and chronic intermittent phencyclidine-induced performance deficits in the 5-choice serial reaction time task: influence of clozapine. *Psychopharmacology* **213**, 681–695 (2011). 20878519 10.1007/s00213-010-2020-7

[19] Dutra-Tavares, A. C. et al. Does nicotine exposure during adolescence modify the course of schizophrenia-like symptoms? Behavioral analysis in a phencyclidine-induced mice model. *Plos One* **16**, e0257986 (2021). 34587208 10.1371/journal.pone.0257986 PMC8480744

[20] Huang, M. et al. Effects of NBI-98782, a selective vesicular monoamine transporter 2 (VMAT2) inhibitor, on neurotransmitter efflux and phencyclidine-induced locomotor activity: Relevance to tardive dyskinesia and antipsychotic action. *Pharmacol., Biochem., Behav.* **190**, 172872 (2020). 32084491 10.1016/j.pbb.2020.172872

[21] Mouri, A. et al. Mouse strain differences in phencyclidine-induced behavioural changes. *Int. J. Neuropsychopharmacol.* **15**, 767–779 (2012). 21733237 10.1017/S146114571100085X

[22] Castane, A., Santana, N. & Artigas, F. PCP-based mice models of schizophrenia: differential behavioral, neurochemical and cellular effects of acute and subchronic treatments. *Psychopharmacology* **232**, 4085–4097 (2015). 25943167 10.1007/s00213-015-3946-6

[23] Turgeon, S. M. & Hoge, S. G. Prior exposure to phencyclidine decreases voluntary sucrose consumption and operant performance for food reward. *Pharmacol., Biochem., Behav.* **76**, 393–400 (2003). 14643837 10.1016/j.pbb.2003.08.019

[24] Lee, P. R., Brady, D. L., Shapiro, R. A., Dorsa, D. M. & Koenig, J. I. Social interaction deficits caused by chronic phencyclidine administration are reversed by oxytocin. *Neuropsychopharmacol. : Off. Publ. Am. Coll. Neuropsychopharmacol.* **30**, 1883–1894 (2005). 10.1038/sj.npp.1300722 15798779

[25] Adams, B. & Moghaddam, B. Corticolimbic dopamine neurotransmission is temporally dissociated from the cognitive and locomotor effects of phencyclidine. *J. Neurosci. : Off. J. Soc. Neurosci.* **18**, 5545–5554 (1998). 10.1523/JNEUROSCI.18-14-05545.1998 PMC6793475 9651235

[26] Jentsch, J. D. et al. Enduring cognitive deficits and cortical dopamine dysfunction in monkeys after long-term administration of phencyclidine. *Sci. (N. Y., N. Y)* **277**, 953–955 (1997). 10.1126/science.277.5328.953 9252326

[27] Broberg, B. V. et al. Assessment of auditory sensory processing in a neurodevelopmental animal model of schizophrenia–gating of auditory-evoked potentials and prepulse inhibition. *Behav. Brain Res.* **213**, 142–147 (2010). 20417666 10.1016/j.bbr.2010.04.026

[28] Savolainen, K., Ihalainen, J., Hamalainen, E., Tanila, H. & Forsberg, M. M. Phencyclidine-induced cognitive impairments in repeated touchscreen visual reversal learning tests in rats. *Behav. Brain Res.* **404**, 113057 (2021). 33316322 10.1016/j.bbr.2020.113057

[29] Shan, L. et al. Schizophrenia-like olfactory dysfunction induced by acute and postnatal phencyclidine exposure in rats. *Schizophr. Res.* **199**, 274–280 (2018). 29510924 10.1016/j.schres.2018.02.045

[30] Mansbach, R. S. & Geyer, M. A. Effects of phencyclidine and phencyclidine biologs on sensorimotor gating in the rat. *Neuropsychopharmacol.: Off. Publ. Am. Coll. Neuropsychopharmacol.* **2**, 299–308 (1989). 10.1016/0893-133X(89)90035-3 2692589

[31] Bialon, M. et al. 1MeTIQ and olanzapine, despite their neurochemical impact, did not ameliorate performance in fear conditioning and social interaction tests in an MK-801 rat model of schizophrenia. *Pharmacol. Rep.: PR* **73**, 490–505 (2021). 33403530 10.1007/s43440-020-00209-9 PMC7994239

[32] Zhan, J.-Q. et al. Flavonoid fisetin reverses impaired hippocampal synaptic plasticity and cognitive function by regulating the function of AMPARs in a male rat model of schizophrenia. *J. Neurochem.* **158**, 413–428 (2021). 33882624 10.1111/jnc.15370

[33] Sawahata, M. et al. Microinjection of Reelin into the mPFC prevents MK-801-induced recognition memory impairment in mice. *Pharm. Res.* **173**, 105832 (2021). 10.1016/j.phrs.2021.105832 34450306

[34] Kozela, E. et al. Cannabidiol Improves Cognitive Impairment and Reverses Cortical Transcriptional Changes Induced by Ketamine, in Schizophrenia-Like Model in Rats. *Mol. Neurobiol.* **57**, 1733–1747 (2020). 31823199 10.1007/s12035-019-01831-2

[35] Azimi Sanavi, M., Ghazvini, H., Zargari, M., Ghalehnoei, H. & Hosseini-Khah, Z. Effects of clozapine and risperidone antipsychotic drugs on the expression of CACNA1C and behavioral changes in rat ‘Ketamine model of schizophrenia. *Neurosci. Lett.* **770**, 136354 (2022). 34801642 10.1016/j.neulet.2021.136354

[36] Sedky, A. A. & Magdy, Y. Reduction in TNF alpha and oxidative stress by liraglutide: Impact on ketamine-induced cognitive dysfunction and hyperlocomotion in rats. *Life Sci.* **278**, 119523 (2021). 33891942 10.1016/j.lfs.2021.119523

[37] Fujikawa, R., Yamada, J. & Jinno, S. Subclass imbalance of parvalbumin-expressing GABAergic neurons in the hippocampus of a mouse ketamine model for schizophrenia, with reference to perineuronal nets. *Schizophr. Res.* **229**, 80–93 (2021). 33229224 10.1016/j.schres.2020.11.016

[38] Perdikaris, P. & Dermon, C. R. Behavioral and neurochemical profile of MK-801 adult zebrafish model: Forebrain beta2-adrenoceptors contribute to social withdrawal and anxiety-like behavior. *Prog. Neuro-Psychoph* **115**, 110494 (2022). 10.1016/j.pnpbp.2021.110494 34896197

[39] Oliveira, A. W. C. et al. Scopolamine and MK-801 impair recognition memory in a new spontaneous object exploration task in monkeys. *Pharmacol., Biochem., Behav.* **211**, 173300 (2021). 34798097 10.1016/j.pbb.2021.173300

[40] Seillier, A. & Giuffrida, A. Evaluation of NMDA receptor models of schizophrenia: divergences in the behavioral effects of sub-chronic PCP and MK-801. *Behav. Brain Res* **204**, 410–415 (2009). 19716985 10.1016/j.bbr.2009.02.007

[41] Wu, B. et al. Prolonged deficits of associative motor learning in cynomolgus monkeys after long-term administration of phencyclidine. *Behav. Brain Res* **331**, 169–176 (2017). 28549649 10.1016/j.bbr.2017.05.035

[42] Thomas, C. G., Miller, A. J. & Westbrook, G. L. Synaptic and extrasynaptic NMDA receptor NR2 subunits in cultured hippocampal neurons. *J. Neurophysiol.* **95**, 1727–1734 (2006). 16319212 10.1152/jn.00771.2005

[43] Huang, H. et al. The potential of the P2X7 receptor as a therapeutic target in a sub-chronic PCP-induced rodent model of schizophrenia. *J. Chem. Neuroanat.* **116**, 101993 (2021). 34147620 10.1016/j.jchemneu.2021.101993

[44] Gigg, J., McEwan, F., Smausz, R., Neill, J. & Harte, M. K. Synaptic biomarker reduction and impaired cognition in the sub-chronic PCP mouse model for schizophrenia. *J. Psychopharmacol. (Oxf., Engl.)* **34**, 115–124 (2020). 10.1177/0269881119874446 31580184

[45] Seillier, A., Martinez, A. A. & Giuffrida, A. Differential effects of Delta9-tetrahydrocannabinol dosing on correlates of schizophrenia in the sub-chronic PCP rat model. *Plos One* **15**, e0230238 (2020). 32163506 10.1371/journal.pone.0230238 PMC7067407

[46] Morris, B. J., Cochran, S. M. & Pratt, J. A. PCP: from pharmacology to modelling schizophrenia. *Curr. Opin. Pharm.* **5**, 101–106 (2005). 10.1016/j.coph.2004.08.008 15661633

[47] Yonezawa, Y., Kuroki, T., Kawahara, T., Tashiro, N. & Uchimura, H. Involvement of gamma-aminobutyric acid neurotransmission in phencyclidine-induced dopamine release in the medial prefrontal cortex. *Eur. J. Pharm.* **341**, 45–56 (1998). 10.1016/S0014-2999(97)01435-0 9489855

[48] Kehr, J. et al. Effects of cariprazine on extracellular levels of glutamate, GABA, dopamine, noradrenaline and serotonin in the medial prefrontal cortex in the rat phencyclidine model of schizophrenia studied by microdialysis and simultaneous recordings of locomotor activity. *Psychopharmacology* **235**, 1593–1607 (2018). 29637288 10.1007/s00213-018-4874-z PMC5920013

[49] Moghaddam, B. & Adams, B. W. Reversal of phencyclidine effects by a group II metabotropic glutamate receptor agonist in rats. *Sci. (N. Y., N. Y)* **281**, 1349–1352 (1998). 10.1126/science.281.5381.1349 9721099

[50] Lorrain, D. S., Baccei, C. S., Bristow, L. J., Anderson, J. J. & Varney, M. A. Effects of ketamine and N-methyl-D-aspartate on glutamate and dopamine release in the rat prefrontal cortex: modulation by a group II selective metabotropic glutamate receptor agonist LY379268. *Neuroscience* **117**, 697–706 (2003). 12617973 10.1016/S0306-4522(02)00652-8

[51] Amargos-Bosch, M., Lopez-Gil, X., Artigas, F. & Adell, A. Clozapine and olanzapine, but not haloperidol, suppress serotonin efflux in the medial prefrontal cortex elicited by phencyclidine and ketamine. *Int J. Neuropsychopharmacol.* **9**, 565–573 (2006). 16316487 10.1017/S1461145705005900

[52] Nelson, C. L., Burk, J. A., Bruno, J. P. & Sarter, M. Effects of acute and repeated systemic administration of ketamine on prefrontal acetylcholine release and sustained attention performance in rats. *Psychopharmacology* **161**, 168–179 (2002). 11981597 10.1007/s00213-002-1004-7

[53] Coyle, J. T. Schizophrenia: Basic and Clinical. *Adv. Neurobiol.* **15**, 255–280 (2017). 28674984 10.1007/978-3-319-57193-5_9

[54] Lin, C.-H., Chen, Y.-M. & Lane, H.-Y. Novel Treatment for the Most Resistant Schizophrenia: Dual Activation of NMDA Receptor and Antioxidant. *Curr. drug targets* **21**, 610–615 (2020). 31660823 10.2174/1389450120666191011163539

[55] Lin, C.-H. & Lane, H.-Y. Early Identification and Intervention of Schizophrenia: Insight From Hypotheses of Glutamate Dysfunction and Oxidative Stress. *Front Psychiatry* **10**, 93 (2019). 30873052 10.3389/fpsyt.2019.00093 PMC6400883

[56] Nakazawa, K. & Sapkota, K. The origin of NMDA receptor hypofunction in schizophrenia. *Pharm. Therapeut* **205**, 107426 (2020). 10.1016/j.pharmthera.2019.107426 PMC6981256 31629007

[57] Tan, Y. et al. Phencyclidine-induced cognitive deficits in mice are ameliorated by subsequent repeated intermittent administration of (R)-ketamine, but not (S)-ketamine: Role of BDNF-TrkB signaling. *Pharmacol., Biochem., Behav.* **188**, 172839 (2020). 31866390 10.1016/j.pbb.2019.172839

[58] Snigdha, S. et al. Phencyclidine (PCP)-induced disruption in cognitive performance is gender-specific and associated with a reduction in brain-derived neurotrophic factor (BDNF) in specific regions of the female rat brain. *J. Mol. Neurosci.: MN* **43**, 337–345 (2011). 20852970 10.1007/s12031-010-9447-5 PMC3041899

[59] Li, Y.-X., Ye, Z.-H., Chen, T., Jia, X.-F. & He, L. The effects of donepezil on phencyclidine-induced cognitive deficits in a mouse model of schizophrenia. *Pharmacol., Biochem., Behav.* **175**, 69–76 (2018). 30218672 10.1016/j.pbb.2018.09.006

[60] Man, L. et al. Cognitive impairments and low BDNF serum levels in first-episode drug-naive patients with schizophrenia. *Psychiatr Res.* **263**, 1–6 (2018). 10.1016/j.psychres.2018.02.034 29482040

[61] Yang, Y. et al. Brain-derived neurotrophic factor is associated with cognitive impairments in first-episode and chronic schizophrenia. *Psychiatr. Res.* **273**, 528–536 (2019). 10.1016/j.psychres.2019.01.051 30710808

[62] Zhang, X. Y. et al. Low BDNF is associated with cognitive impairment in chronic patients with schizophrenia. *Psychopharmacology* **222**, 277–284 (2012). 22274000 10.1007/s00213-012-2643-y

[63] Zhang, Y. et al. Brain-derived neurotrophic factor as a biomarker for cognitive recovery in acute schizophrenia: 12-week results from a prospective longitudinal study. *Psychopharmacology* **235**, 1191–1198 (2018). 29392373 10.1007/s00213-018-4835-6

[64] Chen, Y., Li, S., Zhang, T., Yang, F. & Lu, B. Corticosterone antagonist or TrkB agonist attenuates schizophrenia-like behavior in a mouse model combining Bdnf-e6 deficiency and developmental stress. *Iscience* **25**, 104609 (2022). 35789832 10.1016/j.isci.2022.104609 PMC9250029

[65] Grace, A. A. Ventral hippocampus, interneurons, and schizophrenia: a new understanding of the pathophysiology of schizophrenia and its implications for treatment and prevention. *Curr. Dir. Psychol. Sci.* **19**, 232–237 (2010). 10.1177/0963721410378032

[66] Jimenez-Sanchez, L. et al. Activation of AMPA Receptors Mediates the Antidepressant Action of Deep Brain Stimulation of the Infralimbic Prefrontal Cortex. *Cereb. cortex (N. Y., N.Y: 1991)* **26**, 2778–2789 (2016). 10.1093/cercor/bhv133 26088969

[67] Lopez-Gil, X. et al. Role of Serotonin and Noradrenaline in the Rapid Antidepressant Action of Ketamine. *Acs Chem. Neurosci.* **10**, 3318–3326 (2019). 31244055 10.1021/acschemneuro.9b00288

[68] Beard, E., Lengacher, S., Dias, S., Magistretti, P. J. & Finsterwald, C. Astrocytes as Key Regulators of Brain Energy Metabolism: New Therapeutic Perspectives. *Front Physiol.* **12**, 825816 (2021). 35087428 10.3389/fphys.2021.825816 PMC8787066

[69] Fattorini, G. et al. GLT-1 expression and Glu uptake in rat cerebral cortex are increased by phencyclidine. *Glia* **56**, 1320–1327 (2008). 18615569 10.1002/glia.20700

[70] Kondziella, D. et al. Glial-neuronal interactions are impaired in the schizophrenia model of repeated MK801 exposure. *Neuropsychopharmacol.: Off. Publ. Am. Coll. Neuropsychopharmacol.* **31**, 1880–1887 (2006). 10.1038/sj.npp.1300993 16395297

[71] Cahill, M. K. et al. Network-level encoding of local neurotransmitters in cortical astrocytes. *Nature* **629**, 146–153 (2024). 38632406 10.1038/s41586-024-07311-5 PMC11062919

[72] Mu, Y. et al. Glia Accumulate Evidence that Actions Are Futile and Suppress Unsuccessful Behavior. *Cell* **178**, 27–43.e19 (2019). 31230713 10.1016/j.cell.2019.05.050

[73] Ma, Z., Stork, T., Bergles, D. E. & Freeman, M. R. Neuromodulators signal through astrocytes to alter neural circuit activity and behaviour. *Nature* **539**, 428–432 (2016). 27828941 10.1038/nature20145 PMC5161596

[74] Katz, M. et al. Glutamate spillover in C. elegans triggers repetitive behavior through presynaptic activation of MGL-2/mGluR5. *Nat. Commun.* **10**, 1882 (2019). 31015396 10.1038/s41467-019-09581-4 PMC6478929

[75] Bindocci, E. et al. Three-dimensional Ca^2+^ imaging advances understanding of astrocyte biology. *Science (New York, N.Y.)* **356** (2017). 10.1126/science.aai8185 28522470

[76] Di Castro, M. A. et al. Local Ca^2+^ detection and modulation of synaptic release by astrocytes. *Nat. Neurosci.* **14**, 1276–1284 (2011). 21909085 10.1038/nn.2929

[77] Wang, Y. et al. Accurate quantification of astrocyte and neurotransmitter fluorescence dynamics for single-cell and population-level physiology. *Nat. Neurosci.* **22**, 1936–1944 (2019). 31570865 10.1038/s41593-019-0492-2 PMC6858541

[78] Paukert, M. et al. Norepinephrine controls astroglial responsiveness to local circuit activity. *Neuron* **82**, 1263–1270 (2014). 24945771 10.1016/j.neuron.2014.04.038 PMC4080721

[79] Guttenplan, K. A. et al. Neurotoxic reactive astrocytes induce cell death via saturated lipids. *Nature* **599**, 102–107 (2021). 34616039 10.1038/s41586-021-03960-y PMC12054010

[80] Zhang, L. et al. Alleviating symptoms of neurodegenerative disorders by astrocyte-specific overexpression of TMEM164 in mice. *Nat. Metab.* **5**, 1787–1802 (2023). 37679556 10.1038/s42255-023-00887-8

[81] Zhu, S. et al. Chronic phencyclidine induces inflammatory responses and activates GSK3beta in mice. *Neurochem Res* **39**, 2385–2393 (2014). 25270429 10.1007/s11064-014-1441-9

[82] He, J. et al. Chronic administration of quetiapine attenuates the phencyclidine-induced recognition memory impairment and hippocampal oxidative stress in rats. *Neuroreport* **29**, 1099–1103 (2018). 30036204 10.1097/WNR.0000000000001078

[83] Tran, H.-Q. et al. Clozapine attenuates mitochondrial burdens and abnormal behaviors elicited by phencyclidine in mice via inhibition of p47 phox; Possible involvements of phosphoinositide 3-kinase/Akt signaling. *J. Psychopharmacol. (Oxf., Engl.)* **32**, 1233–1251 (2018). 10.1177/0269881118795244 30207504

[84] Ohnishi, T. et al. Investigation of betaine as a novel psychotherapeutic for schizophrenia. *Ebiomedicine* **45**, 432–446 (2019). 31255657 10.1016/j.ebiom.2019.05.062 PMC6642071

[85] Gundlach, A. L., Largent, B. L. & Snyder, S. H. Phencyclidine (PCP) receptors: autoradiographic localization in brain with the selective ligand, [3H]TCP. *Brain Res.* **386**, 266–279 (1986). 3022881 10.1016/0006-8993(86)90163-0

[86] Maragos, W. F., Greenamyre, J. T., Chu, D. C., Penney, J. B. & Young, A. B. A study of cortical and hippocampal NMDA and PCP receptors following selective cortical and subcortical lesions. *Brain Res* **538**, 36–45 (1991). 1850317 10.1016/0006-8993(91)90373-4

[87] Weinberger, D. R. Cell biology of the hippocampal formation in schizophrenia. *Biol. Psychiatr.* **45**, 395–402 (1999). 10.1016/S0006-3223(98)00331-X 10071707

[88] Jodo, E. et al. Activation of medial prefrontal cortex by phencyclidine is mediated via a hippocampo-prefrontal pathway. *Cereb. cortex (N.Y., N.Y. : 1991)* **15**, 663–669 (2005). 10.1093/cercor/bhh168 15342431

[89] Aghajanian, G. K. & Marek, G. J. Serotonin model of schizophrenia: emerging role of glutamate mechanisms. *Brain Res. Brain Res. Rev.* **31**, 302–312 (2000). 10719157 10.1016/S0165-0173(99)00046-6

[90] Suzuki, Y., Jodo, E., Takeuchi, S., Niwa, S. & Kayama, Y. Acute administration of phencyclidine induces tonic activation of medial prefrontal cortex neurons in freely moving rats. *Neuroscience* **114**, 769–779 (2002). 12220577 10.1016/S0306-4522(02)00298-1

[91] Kargieman, L., Santana, N., Mengod, G., Celada, P. & Artigas, F. Antipsychotic drugs reverse the disruption in prefrontal cortex function produced by NMDA receptor blockade with phencyclidine. *P Natl Acad. Sci. USA* **104**, 14843–14848 (2007). 10.1073/pnas.0704848104 PMC1976198 17785415

[92] Lopez-Gil, X. et al. Importance of inter-hemispheric prefrontal connection in the effects of non-competitive NMDA receptor antagonists. *Int. J. Neuropsychopharmacol.* **15**, 945–956 (2012). 21733285 10.1017/S1461145711001064

[93] Llado-Pelfort, L. et al. Effects of Hallucinogens on Neuronal Activity. *Curr. Top. Behav. Neurosci.* **36**, 75–105 (2018). 28238186 10.1007/7854_2017_473

[94] Katayama, T. et al. Activation of medial prefrontal cortex neurons by phencyclidine is mediated via AMPA/kainate glutamate receptors in anesthetized rats. *Neuroscience* **150**, 442–448 (2007). 17935894 10.1016/j.neuroscience.2007.09.007

[95] Homayoun, H., Jackson, M. E. & Moghaddam, B. Activation of metabotropic glutamate 2/3 receptors reverses the effects of NMDA receptor hypofunction on prefrontal cortex unit activity in awake rats. *J. Neurophysiol.* **93**, 1989–2001 (2005). 15590730 10.1152/jn.00875.2004

[96] Jodo, E. The role of the hippocampo-prefrontal cortex system in phencyclidine-induced psychosis: a model for schizophrenia. *J. Physiol., Paris* **107**, 434–440 (2013). 23792022 10.1016/j.jphysparis.2013.06.002

[97] Perez, S. M., Shah, A., Asher, A. & Lodge, D. J. Hippocampal deep brain stimulation reverses physiological and behavioural deficits in a rodent model of schizophrenia. *Int. J. Neuropsychopharmacol.* **16**, 1331–1339 (2013). 23190686 10.1017/S1461145712001344 PMC3685478

[98] Callicott, J. H. et al. Complexity of prefrontal cortical dysfunction in schizophrenia: more than up or down. *Am. J. Psychiatry* **160**, 2209–2215, (2003). 14638592 10.1176/appi.ajp.160.12.2209

[99] Barch, D. M., Sheline, Y. I., Csernansky, J. G. & Snyder, A. Z. Working memory and prefrontal cortex dysfunction: specificity to schizophrenia compared with major depression. *Biol. Psychiatr.* **53**, 376–384 (2003). 10.1016/S0006-3223(02)01674-8 12614990

[100] Bikovsky, L. et al. Deep brain stimulation improves behavior and modulates neural circuits in a rodent model of schizophrenia. *Exp. Neurol.* **283**, 142–150 (2016). 27302677 10.1016/j.expneurol.2016.06.012 PMC5319857

[101] Jessen, F. et al. Reduced hippocampal activation during encoding and recognition of words in schizophrenia patients. *Am. J.Psychiatry* **160**, 1305–1312 (2003). 12832246 10.1176/appi.ajp.160.7.1305

[102] Ewing, S. G. & Winter, C. The ventral portion of the CA1 region of the hippocampus and the prefrontal cortex as candidate regions for neuromodulation in schizophrenia. *Med. Hypotheses* **80**, 827–832 (2013). 23583328 10.1016/j.mehy.2013.03.026

[103] Miller, E. J., Saint Marie, L. R., Breier, M. R. & Swerdlow, N. R. Pathways from the ventral hippocampus and caudal amygdala to forebrain regions that regulate sensorimotor gating in the rat. *Neuroscience* **165**, 601–611 (2010). 19854244 10.1016/j.neuroscience.2009.10.036 PMC2796367

[104] Sotres-Bayon, F., Sierra-Mercado, D., Pardilla-Delgado, E. & Quirk, G. J. Gating of fear in prelimbic cortex by hippocampal and amygdala inputs. *Neuron* **76**, 804–812 (2012). 23177964 10.1016/j.neuron.2012.09.028 PMC3508462

[105] Dienel, S. J., Enwright, J. F. 3rd, Hoftman, G. D. & Lewis, D. A. Markers of glutamate and GABA neurotransmission in the prefrontal cortex of schizophrenia subjects: Disease effects differ across anatomical levels of resolution. *Schizophr. Res* **217**, 86–94 (2020). 31296415 10.1016/j.schres.2019.06.003 PMC6946893

[106] Abdallah, C. G. et al. The effects of ketamine on prefrontal glutamate neurotransmission in healthy and depressed subjects. *Neuropsychopharmacol.: Off. Publ. Am. Coll. Neuropsychopharmacol.* **43**, 2154–2160 (2018). 10.1038/s41386-018-0136-3 PMC6098048 29977074

[107] Fan, Z.-L. et al. Optogenetic inhibition of ventral hippocampal neurons alleviates associative motor learning dysfunction in a rodent model of schizophrenia. *Plos One* **14**, e0227200 (2019). 31891640 10.1371/journal.pone.0227200 PMC6938361

[108] Grunze, H. C. et al. NMDA-dependent modulation of CA1 local circuit inhibition. *J. Neurosci.: Off. J. Soc. Neurosci.* **16**, 2034–2043 (1996). 10.1523/JNEUROSCI.16-06-02034.1996 PMC6578507 8604048

[109] Freund, T. F. & Katona, I. Perisomatic inhibition. *Neuron* **56**, 33–42 (2007). 17920013 10.1016/j.neuron.2007.09.012

[110] Monyer, H., Burnashev, N., Laurie, D. J., Sakmann, B. & Seeburg, P. H. Developmental and regional expression in the rat brain and functional properties of four NMDA receptors. *Neuron* **12**, 529–540 (1994). 7512349 10.1016/0896-6273(94)90210-0

[111] Straub, R. E. et al. Allelic variation in GAD1 (GAD67) is associated with schizophrenia and influences cortical function and gene expression. *Mol. Psychiatr.* **12**, 854–869 (2007). 10.1038/sj.mp.4001988 17767149

[112] Kumari, V., Soni, W., Mathew, V. M. & Sharma, T. Prepulse inhibition of the startle response in men with schizophrenia: effects of age of onset of illness, symptoms, and medication. *Arch. Gen. Psychiatr.* **57**, 609–614 (2000). 10839340 10.1001/archpsyc.57.6.609

[113] Kocsis, B. Differential role of NR2A and NR2B subunits in N-methyl-D-aspartate receptor antagonist-induced aberrant cortical gamma oscillations. *Biol. Psychiatr.* **71**, 987–995 (2012). 10.1016/j.biopsych.2011.10.002 PMC3276718 22055014

[114] Sullivan, E. M., Timi, P., Hong, L. E. & O’Donnell, P. Reverse translation of clinical electrophysiological biomarkers in behaving rodents under acute and chronic NMDA receptor antagonism. *Neuropsychopharmacol.: Off. Publ. Am. Coll. Neuropsychopharmacol.* **40**, 719–727 (2015). 10.1038/npp.2014.228 PMC4289960 25176166

[115] Noda, Y., Kamei, H., Mamiya, T., Furukawa, H. & Nabeshima, T. Repeated phencyclidine treatment induces negative symptom-like behavior in forced swimming test in mice: imbalance of prefrontal serotonergic and dopaminergic functions. *Neuropsychopharmacol. : Off. Publ. Am. Coll. Neuropsychopharmacol.* **23**, 375–387 (2000). 10.1016/S0893-133X(00)00138-X 10989264

[116] Nath, M., Bhardwaj, S. K., Srivastava, L. K. & Wong, T. P. Altered excitatory and decreased inhibitory transmission in the prefrontal cortex of male mice with early developmental disruption to the ventral hippocampus. *Cereb. cortex (N. Y., N.Y: 1991)* **33**, 865–880 (2023). 10.1093/cercor/bhac107 PMC9890473 35297476

[117] Gaskin, P. L., Toledo-Rodriguez, M., Alexander, S. P. & Fone, K. C. Down-regulation of hippocampal genes regulating dopaminergic, GABAergic, and glutamatergic function following combined neonatal phencyclidine and post-weaning social isolation of rats as a neurodevelopmental model for schizophrenia. *Int. J. Neuropsychopharmacol.* **19**, pyw062 (2016). 27382048 10.1093/ijnp/pyw062 PMC5137279

[118] Hervig, M. E., Thomsen, M. S., Kallo, I. & Mikkelsen, J. D. Acute phencyclidine administration induces c-Fos-immunoreactivity in interneurons in cortical and subcortical regions. *Neuroscience* **334**, 13–25 (2016). 27476436 10.1016/j.neuroscience.2016.07.028

[119] Santana, N., Troyano-Rodriguez, E., Mengod, G., Celada, P. & Artigas, F. Activation of thalamocortical networks by the N-methyl-D-aspartate receptor antagonist phencyclidine: reversal by clozapine. *Biol. Psychiatr.* **69**, 918–927 (2011). 10.1016/j.biopsych.2010.10.030 21251645

[120] Troyano-Rodriguez, E. et al. Phencyclidine inhibits the activity of thalamic reticular gamma-aminobutyric acidergic neurons in rat brain. *Biol. Psychiatr.* **76**, 937–945 (2014). 10.1016/j.biopsych.2014.05.019 25038984

[121] Jodo, E. et al. Differences in responsiveness of mediodorsal thalamic and medial prefrontal cortical neurons to social interaction and systemically administered phencyclidine in rats. *Neuroscience* **170**, 1153–1164 (2010). 20727386 10.1016/j.neuroscience.2010.08.017

[122] Kuroda, M., Yokofujita, J. & Murakami, K. An ultrastructural study of the neural circuit between the prefrontal cortex and the mediodorsal nucleus of the thalamus. *Prog. Neurobiol.* **54**, 417–458 (1998). 9522395 10.1016/S0301-0082(97)00070-1

[123] Bubser, M. et al. Disinhibition of the mediodorsal thalamus induces fos-like immunoreactivity in both pyramidal and GABA-containing neurons in the medial prefrontal cortex of rats, but does not affect prefrontal extracellular GABA levels. *Synap. (N. Y., N. Y.)* **30**, 156–165 (1998). 10.1002/(SICI)1098-2396(199810)30:2<156::AID-SYN5>3.0.CO;2-B 9723785

[124] Floresco, S. B. & Grace, A. A. Gating of hippocampal-evoked activity in prefrontal cortical neurons by inputs from the mediodorsal thalamus and ventral tegmental area. *J. Neurosci. : Off. J. Soc. Neurosci.* **23**, 3930–3943 (2003). 10.1523/JNEUROSCI.23-09-03930.2003 PMC6742171 12736363

[125] Grayson, B. et al. Postnatal Phencyclidine (PCP) as a Neurodevelopmental Animal Model of Schizophrenia Pathophysiology and Symptomatology: A Review. *Curr. Top. Behav. Neurosci.* **29**, 403–428 (2016). 26510740 10.1007/7854_2015_403

[126] Moghadam, A. A., Vose, L. R., Miry, O., Zhang, X.-L. & Stanton, P. K. Pairing of neonatal phencyclidine exposure and acute adolescent stress in male rats as a novel developmental model of schizophrenia. *Behav. Brain Res* **409**, 113308 (2021). 33872663 10.1016/j.bbr.2021.113308

[127] Feigenson, K. A., Kusnecov, A. W. & Silverstein, S. M. Inflammation and the two-hit hypothesis of schizophrenia. *Neurosci. Biobehav R.* **38**, 72–93 (2014). 10.1016/j.neubiorev.2013.11.006 PMC3896922 24247023

[128] Gomes, F. V., Rincon-Cortes, M. & Grace, A. A. Adolescence as a period of vulnerability and intervention in schizophrenia: Insights from the MAM model. *Neurosci. Biobehav R.* **70**, 260–270 (2016). 10.1016/j.neubiorev.2016.05.030 PMC5074867 27235082

[129] Murray, R. M., Bhavsar, V., Tripoli, G. & Howes, O. 30 Years on: How the Neurodevelopmental Hypothesis of Schizophrenia Morphed Into the Developmental Risk Factor Model of Psychosis. *Schizophrenia Bull.* **43**, 1190–1196 (2017). 10.1093/schbul/sbx121 PMC5737804 28981842

[130] Selemon, L. D. & Zecevic, N. Schizophrenia: a tale of two critical periods for prefrontal cortical development. *Transl. Psychiat.* **5**, e623 (2015). 10.1038/tp.2015.115 PMC4564568 26285133

[131] Kalopita, K., Armakolas, A., Philippou, A., Zarros, A. & Angelogianni, P. Ketamine-induced neurotoxicity in neurodevelopment: A synopsis of main pathways based on recent in vivo experimental findings. *J. Anaesthesiol., Clin. Pharmacol.* **37**, 37–42 (2021). 34103820 10.4103/joacp.JOACP_415_19 PMC8174420

[132] Pratt, J., Winchester, C., Dawson, N. & Morris, B. Advancing schizophrenia drug discovery: optimizing rodent models to bridge the translational gap. *Nat. Rev. Drug Discov.* **11**, 560–579 (2012). 22722532 10.1038/nrd3649

[133] Goff, D. C. & Coyle, J. T. The emerging role of glutamate in the pathophysiology and treatment of schizophrenia. *Am. J. psychiatry* **158**, 1367–1377 (2001). 11532718 10.1176/appi.ajp.158.9.1367

[134] Javitt, D. C. Glutamatergic theories of schizophrenia. *Isr. J. Psychiatry Relat. Sci.* **47**, 4–16 (2010). 20686195

[135] Hanson, J. E. et al. Therapeutic potential of N-methyl-D-aspartate receptor modulators in psychiatry. *Neuropsychopharmacol.: Off. Publ. Am. Coll. Neuropsychopharmacol.* **49**, 51–66 (2024). 10.1038/s41386-023-01614-3 PMC10700609 37369776

[136] Zheng, W. et al. Adjunctive memantine for schizophrenia: a meta-analysis of randomized, double-blind, placebo-controlled trials. *Psychol. Med.* **48**, 72–81 (2018). 28528597 10.1017/S0033291717001271

[137] Zheng, W. et al. Adjunctive memantine for major mental disorders: A systematic review and meta-analysis of randomized double-blind controlled trials. *Schizophr. Res.* **209**, 12–21 (2019). 31164254 10.1016/j.schres.2019.05.019

[138] Schaefer, M. et al. Acute and Long-term Memantine Add-on Treatment to Risperidone Improves Cognitive Dysfunction in Patients with Acute and Chronic Schizophrenia. *Pharmacopsychiatry* **53**, 21–29 (2020). 31390660 10.1055/a-0970-9310

[139] Magdaleno-Madrigal, V. M. et al. Short-term deep brain stimulation of the thalamic reticular nucleus modifies aberrant oscillatory activity in a neurodevelopment model of schizophrenia. *Neuroscience* **357**, 99–109 (2017). 28576730 10.1016/j.neuroscience.2017.05.035

[140] Ewing, S. G. & Grace, A. A. Deep brain stimulation of the ventral hippocampus restores deficits in processing of auditory evoked potentials in a rodent developmental disruption model of schizophrenia. *Schizophr. Res* **143**, 377–383 (2013). 23269227 10.1016/j.schres.2012.11.023 PMC3547127

[141] Corripio, I. et al. Clinical improvement in a treatment-resistant patient with schizophrenia treated with deep brain stimulation. *Biol. Psychiat.* **80**, e69–70 (2016). 27113497 10.1016/j.biopsych.2016.03.1049

[142] Zain, M. A. et al. Phencyclidine dose optimisation for induction of spatial learning and memory deficits related to schizophrenia in C57BL/6 mice. *Exp. Anim. Tokyo* **67**, 421–429 (2018). 10.1538/expanim.18-0006 PMC6219884 29731492

